# Ischemia Elicits a Coordinated Expression of Pro-Survival Proteins in Mouse Myocardium

**DOI:** 10.1100/tsw.2002.192

**Published:** 2002-04-11

**Authors:** Deborah Lyn, Shaojia Bao, Nicole A. Bennett, Xiaowei Liu, Nerimiah L. Emmett

**Affiliations:** Department of Microbiology, Biochemistry and Immunology, Morehouse School of Medicine, 720 Westview Drive, Atlanta, GA 30310, USA; Department of Physiology, Morehouse School of Medicine, 720 Westview Drive, Atlanta, GA 30310, USA

**Keywords:** ischemia, cardiac, apoptosis, mouse, pro-survival

## Abstract

Cardiomyocytes are post-mitotic, long-lived cells until disruptions to pro-survival factors occur after myocardial ischemia. To gain an understanding of the factors involved with ischemic injury, we examined expression changes in pro-survival and opposing pro-apoptotic signals at early and chronic periods of ischemia using an in vivo murine model. Alterations of pro-survival proteins such as the inhibitor of apoptosis protein on chromosome X (xIAP) and the apoptotic repressor protein (ARC) have not been evaluated in a murine model of cardiac ischemia. Early ischemia (1 day) resulted in a 50% reduction in ARC protein levels relative to sham-operated left ventricles, without significant changes in the expression of xIAP or other pro-survival factors. In contrast, a deficiency of xIAP expression was found in cardiac infarcts starting after 1 week, concomitant with significant evidence of apoptotic cell death and an up-regulation of pro-apoptotic signals including Bax, tumor necrosis factor-a, and caspase-8 activation. Chronic ischemia (after 2 weeks) was associated with elevated levels of other pro-survival factors such as Bcl-x_L_ and the phosphorylated form of Akt, as part of the adaptive remodeling of the myocardium. Altogether, these findings suggest that strategies to increase IAP expression may promote myocyte survival after chronic ischemia.

## INTRODUCTION

Cardiomyocytes are post-mitotic long-lived cells until disturbances in cell survival proteins occur as a result of pathological conditions such as myocardial ischemia[1,2,3]. Clinically, a loss of myocytes has been associated with cardiac ischemia leading to the development of heart failure[4]. This complex process involves a network of converging pathways in which cardiomyocyte survival depends on the pro-apoptotic (programmed cell death) process counterbalanced by cell survival factors to limit cellular injury and preserve ventricular function. While various studies have examined apoptosis as an end-point in cardiac ischemia, less attention has been given to analyzing the expression of cell survival proteins after ischemic injury in a mouse model.

The identification of cell survival signals that participate in the disease cascade is critical to understanding the signaling pathways that guide cardiomyocyte survival. A protective role of pro-survival proteins has been demonstrated for the apoptotic repressor Bcl-2 and Akt kinase in cardiomyocytes *in vitro*[5,6,7,8], after gene transfer[9], and in transgenic mice [10,11]. Using a transient ischemic model in conscious pigs, Depre et al.[12] observed an activation of a cell survival program including genes involved in the prevention of apoptosis, cytoprotection, and cell growth. Furthermore, gene expression changes were proportional to the severity of the ischemic insult.

Since the survival of the myocardium after ischemia may depend on opposing pro- and anti-apoptotic signals, we examined the expression of proteins known to interfere with cell death by inhibiting the activity of caspases, cysteine proteases that play a critical role in executing apoptosis[13,14]. The apoptosis-inhibiting protein ARC (apoptosis repressor with CARD [caspase activation recruitment domain]) can inhibit apoptosis *in vitro* by selective interactions with the apical caspases-2 and -8[15,16], while the inhibitor of apoptosis protein (IAP) may directly suppress the activity of caspases-3, -6, -7, and -9[17,18,19]. In this study, we found a differential expression of ARC and IAP in response to acute and chronic ischemia. We also assessed the relative levels of other pro-survival molecules (Bcl-xL, Akt) as well as pro-apoptotic factors (Bax, caspases, Bid, tumor necrosis factor-α, apoptosis inducing factor).

## EXPERIMENTAL METHODS AND PROCEDURES

Cardiac ischemia was induced using an established murine model involving surgical constriction of the left anterior descending coronary artery[20]. Ischemia was induced in adult male ICR mice (Harlan Sprague Dawley) for varying time periods, while sham-operated controls underwent a similar surgical procedure without constriction of the coronary artery. At indicated times of ischemia, heart tissues were collected after inducing anesthesia with ketamine (100 mg/Kg, IM) prior to sacrificing the animal by cervical dislocation.

Protein expression was monitored by western blots and by immunocytochemistry. For western blots, left ventricle tissues were dissected after collection and homogenized in a cold lysis buffer of 150 m*M* NaCl, 10 m*M* sodium phosphate pH 7.4, 1% NP40, 0.1% SDS, 0.5% sodium deoxycholate, 5 m*M* EGTA, 1 m*M* PMFS, 100 μM Nα-p-tosyl-lysine chloro-methyl ketone (TLCK), Sigma protease inhibitor cocktail (50 μl/g tissue). Protein concentration (BioRad DC assay) was determined in lysates after clearing by centrifugation. Extracts were separated by sodium dodecyl sulfate–polyacrylamide gel electrophoresis, transferred to polyvinylidene difluoride membranes and blotted against the indicated antibodies overnight at 4°C. Each lane on the western contained 50 μg protein derived from the left ventricle of one animal. Western blots were independently repeated from ischemic tissues collected from a minimum of four animals at each time point and normalized to glyceraldehyde 3-phosphate dehydrogenase (G3PDH). The relative intensity of the chemiluminescent signal was captured on X-ray film and quantified by densitometry on a Nucleovision system (Nucleotech). Antibodies were obtained from the following sources using recommended dilutions: xIAP from StressGen Biotechnologies Corp.; ARC and caspase-8 from Chemicon; Bcl-$x_L$ and Bax from BD Sciences; phospho$^{Ser473}$Akt, Akt, and BID from Cell Signaling Technology; tumor necrosis factor alpha (TNF-α) from R & D Systems, Inc.; and G3PDH from Research Diagnostics.

Immunocytochemistry was assessed from frozen or fresh tissues, fixed in Bouin's solution, and embedded in paraffin. Sections of 5 μm were subjected to immunostaining, and the antigenantibody complex detected by avidin-biotin-immunoperoxidase followed by exposure to diaminobenzidine (Vectastain Elite ABC kit, Vector Laboratories). Hematoxylin was used as a counter stain. The primary antibody was omitted from negative controls. Sections were collected from three mice of each ischemic time interval, and were also used for examination of apoptosis.

Apoptosis was detected by the ApopTag *in situ* oligo ligation (Intergen), which specifically detects 3' overhang DNA-strand breaks by biotin labeling of hairpin oligonucleotides. This technique can selectively discriminate between apoptotic and necrotic DNA[21]. Negative controls were without the addition of DNA ligase.

Recombinant mouse TNF-α (activity $\geq 1 \times 10^7$ units/mg) at a dose of 600 ng/animal in 0.1 ml PBS (BioSource International) was administered i.v. to the jugular vein under anesthesia. This dose has previously been shown to induce chemokine mRNA expression in the heart[22]. Data were collected from three mice in each experiment.

Data are expressed as means ± SEM, in which different experimental groupings were assessed by analysis of variance (ANOVA). Minimal statistical significance was judged at $p < 0.05$.

## RESULTS AND DISCUSSION

The purpose of this study was to assess the physiological and pathological importance of pro-survival factors during cardiac ischemia. We postulated that ischemia would be associated with a lower expression of pro-survival proteins. Shorter periods of cardiac ischemia (1 day) elicited a different response from chronic time periods (weeks). The apoptotic repressor ARC has a restricted transcription to myocytes, whose expression has not been evaluated in an animal model of cardiac ischemia. Lower protein levels of ARC were observed at acute time periods, but not at chronic times relative to sham-operated tissues (presented in Fig. 1 and summarized in Table 1). Moreover, modifications such as phosphorylation have also been thought to play a role in transmitting the anti-apoptotic effect of ARC[23]. The appearance of active (cleaved) forms of both caspase-8 and its substrate BID (Fig. 1) along with apoptosis in a limited population of myocytes (Fig. 2) accompanied lower ARC expression levels after 24 h, indicating a greater susceptibility to ischemic injury. These results are analogous to studies showing a deficit of

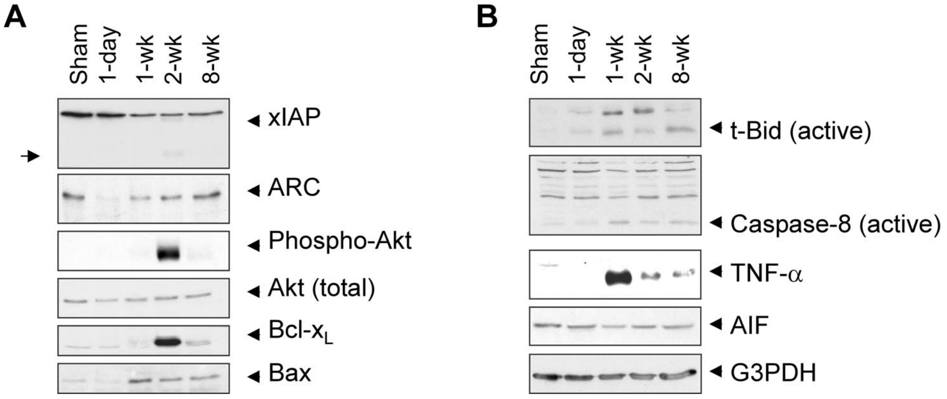

**FIGURE 1.** Western blot analysis of (A) pro-survival and (B) pro-apoptotic proteins after induction of ischemia. Blots were assessed from a minimum of four independently collected tissues. Bax (pro-apoptotic) levels are included for comparison with Bcl $x_L$ (pro-survival). Ratios of Bax:Bcl $x_L$ were 1.1 ± 0.4 (sham), 1.7 ± 0.3 (1 day), 2.8 ± 0.2 (1 week), 0.5 ± 0.2 (2 weeks), 2.0 ± 0.6 (8 weeks). Arrow indicates endogenous cleaved xIAP fragment[29]. Data presented are collected and summarized in Table 1.

**TABLE 1**
**Summary of Protein Expression Changes**

| | Ischemic Time | | |
|---|---|---|---|
| **Protein** | **1 day** | **1 week** | **2 weeks** |
| ARC | 0.47 ± 0.03[2] | 0.83 ± 0.09 | 1.1 ± 0.15 |
| xIAP | 0.90 ± 0.1 | 0.64 ± 0.05[1] | 0.72 ± 0.02[1] |
| Phospho-Akt | Undetected | Undetected | Detected |
| Caspase-8 | 1.40 ± 0.10 | 4.00 ± 0.70[2] | 2.90 ± 0.20[2] |
| t-BID | 2.50 ± 0.40[1] | 7.90 ± 0.80[2] | 4.10 ± 0.50[2] |
| TNF-α | Undetected | Detected | Detected |
| AIF | 0.95 ± 0.20 | 0.88 ± 0.30 | 0.98 ± 0.20 |

Protein levels are derived from western blots and expressed relative to sham-operated controls (1.0). Protein levels at each ischemic time were divided by the expression observed in sham controls.
[1] $p < 0.05$
[2] $p < 0.01$

FLIP/Usurpin expression in rat infarcted cardiac tissue[24]. Similar to ARC[15,16], FLIP/Usurpin represents an anti-apoptotic protein with a caspase activation recruitment domain (CARD) that inhibits cell death via selective interactions with the apical caspases.

Because it has been hypothesized that ARC may disrupt the association between caspase-8 and death adapters triggered by TNF-α *in vitro*[15], we also sought to determine whether this cytokine could down-regulate ARC expression *in vivo*. Moreover, elevated serum levels of TNF-α are also found clinically after myocardial infarction[25,26]. No significant changes in ARC expression were observed over a 2-h time period (1.0 [PBS/control]; 0.97 [1 h]; 1.10 [2 h] based on an average of three mice per group), suggesting that ARC may be modulated by early ischemic stress but not by cytokine-mediated processes. Protein levels of ARC also do not change significantly when cardiac tissue TNF-α levels are elevated.

The greatest degree of apoptosis was observed after 1 week of ischemia (Fig. 2C), which correlated with the highest tissue levels of TNF-α as well as activation of other pro-apoptotic factors[27,28], caspase-8 (fourfold) and t-Bid (eightfold) as shown in Fig. 1B and Table 1. These observations were concomitant with a lower expression (64–72% of protein levels in sham tissue) of the pro-survival molecule IAP (Fig. 1A) located on chromosome X (xIAP). This was further confirmed by immunocytochemistry in which lower xIAP expression was observed in the infarct region and adjacent zones (Fig. 3). We also observed an endogenous cleaved fragment of xIAP of about 30 kDa (more apparent on longer exposures) at time points of ischemia associated with higher levels of tissue TNF-α, consistent with loss of the anti-apoptotic function of IAP [29].

In contrast, chronic ischemia at 2 weeks was associated with an activation of other pro-survival factors Akt/protein kinase B (PKB), assessed by changes in phosphorylation, along with increased Bcl-x protein levels but without any expression changes at shorter intervals of ischemia (Fig. 1A). Evidence of Akt activation has been observed in human hearts failing due to ischemia or idiopathic cardiomyopathy[30]. Collectively, the stimulation of pro-survival factors suggests that compensatory changes can occur during the adaptive remodeling of the myocardium. In this regard, we have observed positive immunocytochemistry staining for class II molecules of the major histocompatibility complex after 2 weeks of ischemia similar to the observed immuno-

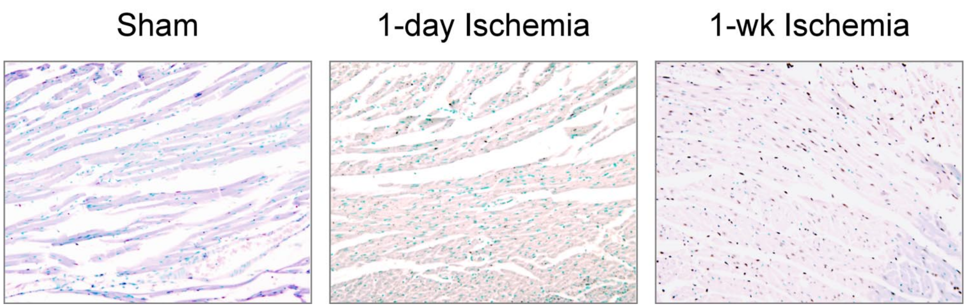

**FIGURE 2.** Assessment of apoptosis by *in situ* oligo ligation. Apoptotic cells were detected by applying streptavidin-peroxidase in the presence of substrate. Sections were counterstained with methyl green. Ischemic tissue represents areas adjacent to the infarct zone.

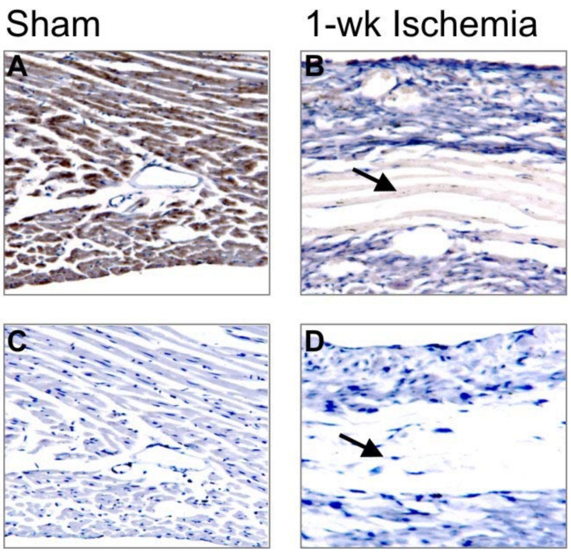

**FIGURE 3.** Immunohistochemical staining with xIAP. Panels A and B represent staining with antibody to xIAP at a dilution of 1:500. Panels C and D were sections from the same tissues without the addition of primary antibody. Arrow indicates the infarcted region. Magnification, X20.

reactivity for Bcl-$x_L$. These observations allude to the possibility that expression of these pro-survival molecules was due to infiltrating leukocytes, neutrophils, and dendritic cells (unpublished observations), associated with an adaptation to ischemic injury including loss of cardiomyocytes and scar deposition[31].

Since an index of susceptibility to cell death may rely on the relative expression levels of other Bcl-2 related proteins[32,33], we also examined protein levels of Bax (Fig. 1A). We observed an increased ratio of Bax:Bcl-x at 1 week of ischemia consistent with transcriptional changes of these molecules[20]. In contrast, ischemia apparently had little effect on apoptosis inducing factor (AIF) protein; see Fig. 1B. However, since AIF translocates from the mitochondrial membrane to the nucleus in response to different death stimuli, its pro-apoptotic role may depend on a sub-cellular redistribution[34,35] that was not detected in cardiac tissues by western blotting. Altogether, a differential expression of pro-survival proteins was dependent on the time period of ischemia. These observations suggest that strategies to increase the expression of apoptotic repressors may enhance myocyte survival after ischemia.

## ACKNOWLEDGEMENTS

This study was supported by NIH grants RR 03034 and GM 08248.
